# First report of pulmonary sclerosing pneomucytoma with malignant transformation in both cuboidal surface cells and stromal round cells: a case report

**DOI:** 10.1186/s12885-019-6356-z

**Published:** 2019-11-27

**Authors:** Xiao Teng, Xiaodong Teng

**Affiliations:** 1 Department of Thoracic Surgery, The First Affiliated Hospital, School of Medicine, Zhejiang University, Qingchun Road 79, Hangzhou, Zhejiang 310003 People’s Republic of China; 2 Department of Pathology, The First Affiliated Hospital, School of Medicine, Zhejiang University, Hangzhou, Zhejiang China

**Keywords:** Pulmonary sclerosing pneomucytoma, Malignant transformation, Mesenchymal to epithelial transition, Stromal round cell, Cuboidal surface cell

## Abstract

**Background:**

Pulmonary sclerosing pneumocytoma (PSP) is a rare benign tumor. Although lymph node metastasis has been reported, it is still considered benign. No malignant transformation has been reported. This is the first case of malignant transformation of both cuboidal surface cells and stromal round cells.

**Case presentation:**

A 64-year-old male had been complaining of intermittent hemoptysis several times per day for eight months. Chest computed tomography scan showed parenchymal infiltration with cystic lesion in the right lower lobe accompanied by enlarged right hilar lymph nodes. Lobectomy and systemic lymph node dissection was performed.

On grossly pathological examination, the lesion was 50 mm from the bronchial stump. It was a mixture of both cystic and solid components and 30 mm * 20 mm in size with unclear border. Microscopically, the cuboidal surface cells transformed to adenocarcinoma. The stromal round cells also had a malignant transformation. The Ki-67 proliferation index in malignant cuboidal surface cells and stromal round cells were 70 and 55%, respectively. Furthermore, E-cadherin was negative in primary tumor but positive in metastatic lymph node, which suggested that the mesenchymal to epithelial transition may play an important role in lymph node metastasis.

**Conclusions:**

To our knowledge, we present the first case of malignant transformation of both cuboidal surface cells and stromal round cells in PSP. The process of mesenchymal to epithelial transition may play an important role in lymph node metastasis.

## Background

Pulmonary sclerosing pneumocytoma (PSP) is a rare benign tumor which has been described as sclerosing hemangioma [1]. It was previously considered as a vascular neoplasm, and now as a derivative from the primitive respiratory epithelium [2]. It is predominant in females, most commonly seen in middle aged females [3, 4]. Patients are always asymptomatic and computed tomography (CT) and X-ray of chest shows solitary, well circumscribed masses. The key pathological features of PSP involve two types of cells, cuboidal surface cells and stromal round cells, which are both neoplastic. Immunohistochemistry (IHC) studies show that thyroid transcription factor-1 (TTF-1) and epithelial membrane antigen (EMA) are both positive [2]. Pancytokeratin (CKpan) and Napsin A are both positive in cuboidal surface cells, while negative in stromal round cells [5]. Though lymph node metastasis has been reported, PSP is still considered benign [6, 7]. We report a unique case of PSP with malignant transformation in both cuboidal surface cells and stromal round cells, which has not been reported before.

## Case presentation

**Fig. 1:**
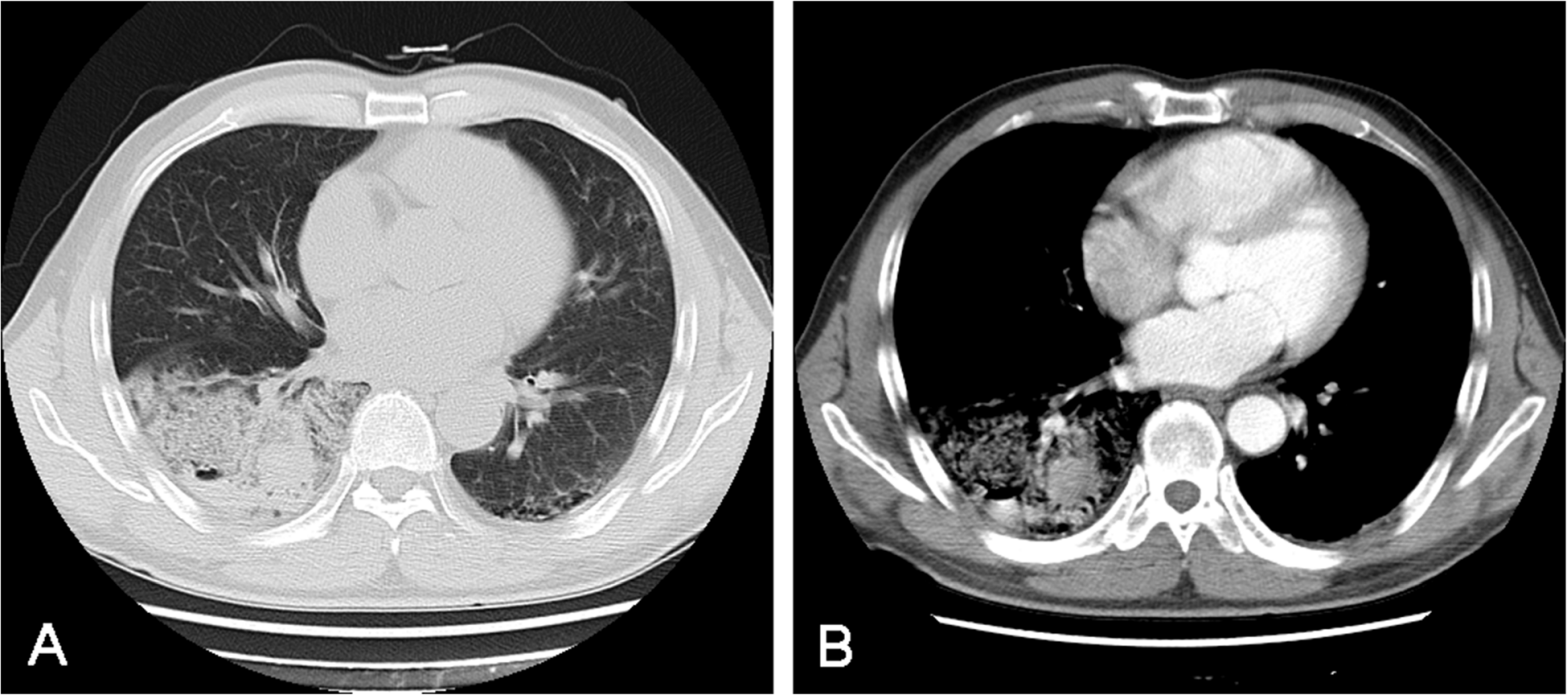
Chest computed tomography scan showed that parenchymal infiltrate with cystic lesion in the right lower lobe of lung

A 64-year-old male had been complaining of intermittent hemoptysis several times per day for eight months. He had no fever, chest pain, shortness of breath, dizziness or amaurosis. He had no relevant medical history especially no history of cancer. He had no smoking history. The patient was admitted to The First Affiliated Hospital, School of Medicine, Zhejiang University due to symptoms getting worse. Chest computed tomography scan on July 1st, 2018 showed parenchymal infiltration with cystic lesion in the right lower lobe accompanied by enlarged right hilar lymph nodes (Fig. 1). Transbronchial lung biopsy under bronchofibroscopy was free of tumor cells. A primary surgical resection was recommended by surgeons. Lobectomy and systemic lymph node dissection was done on July 4th, 2018. The patient is now well after he recovered from surgery. So far there were no signs of tumor recurrence or metastasis.

Upon grossly pathological examination, the lesion was located in the right lower lobe, 50 mm from the bronchial stump. It was gray-tan to yellow on the section, with foci of hemorrhage. The lesion was a mixture of both cystic and solid components and was 30 mm *20 mm in size with unclear border. The solid component was in the middle of the lesion and was 17 mm*17 mm in size, surrounded by honeycomb cystic components.

**Fig. 2:**
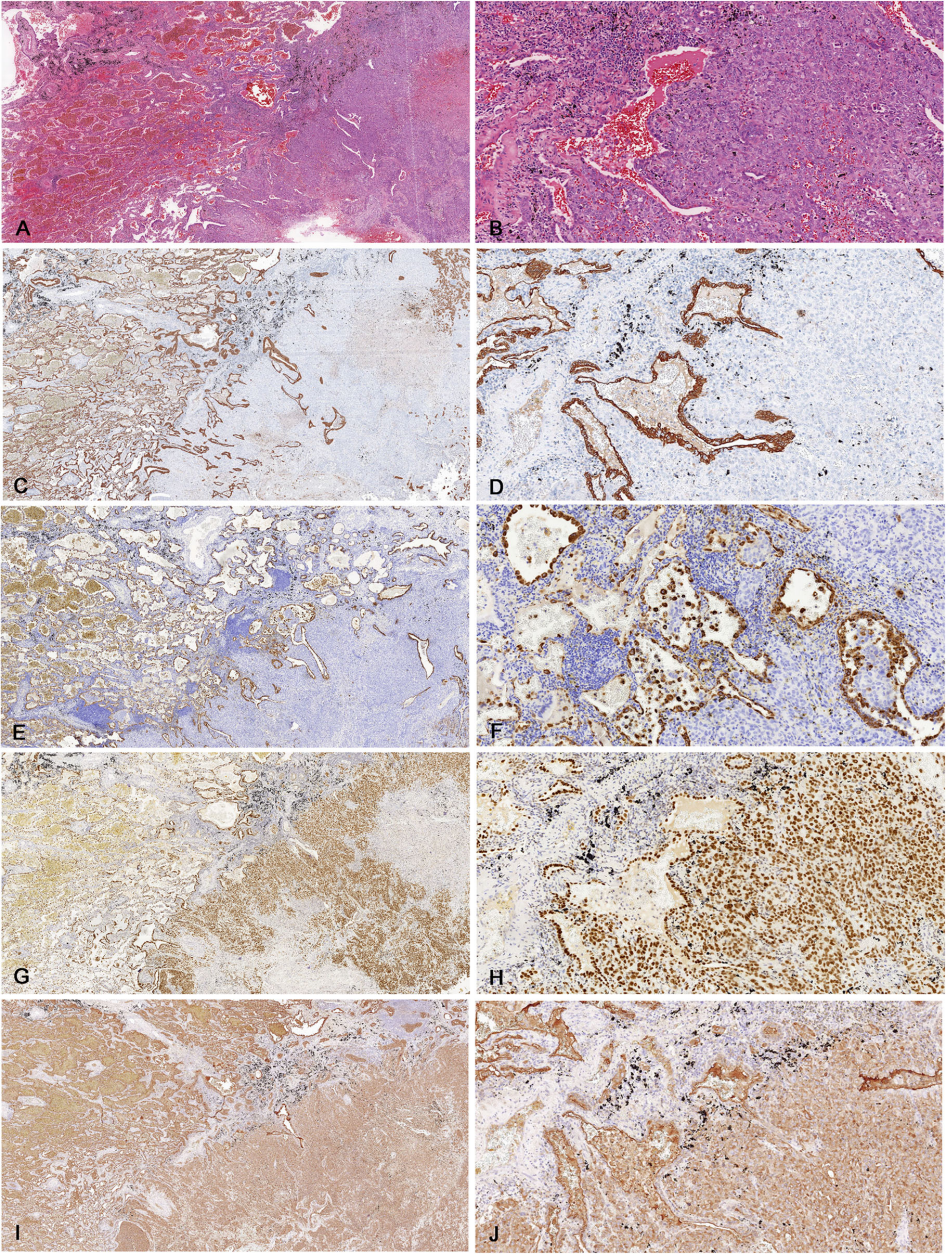
(**a**, **b**) Tumor nodule showed a typical pulmonary sclerosing pneumocytoma of hemorrhage growth pattern comprising of large blood-filled spaces lined by surface cells (**h**&**e**). (**c**, **d**) Cuboidal surface cell were positive for pancytokeratin (CKpan), round cells were negative for CKpan. (**e**, **f**) Cuboidal surface cell were positive for Napsin A, round cells were negative. (**g**, **h**) Both cuboidal surface and stromal round cells were positive for thyroid transcription factor-1 (TTF-1). (**i**, **j**) Both cuboidal surface and stromal round cells were positive for epithelial membrane antigen (EMA)

Microscopically, the structure of the solid component of the tumor was similar to a typical PSP. It was composed of areas of cuboidal surface cells and stromal round cells. The tumor showed a hemorrhage pattern (Fig. 2). Bronchial adenomatous hyperplasia and cystic dilatation were noticed in surrounding areas. TTF-1 and EMA were positive in both cuboidal surface cells and stromal round cells (Fig. 2) while CKpan and Napsin A were only positive in cuboidal surface cells.

**Fig. 3:**
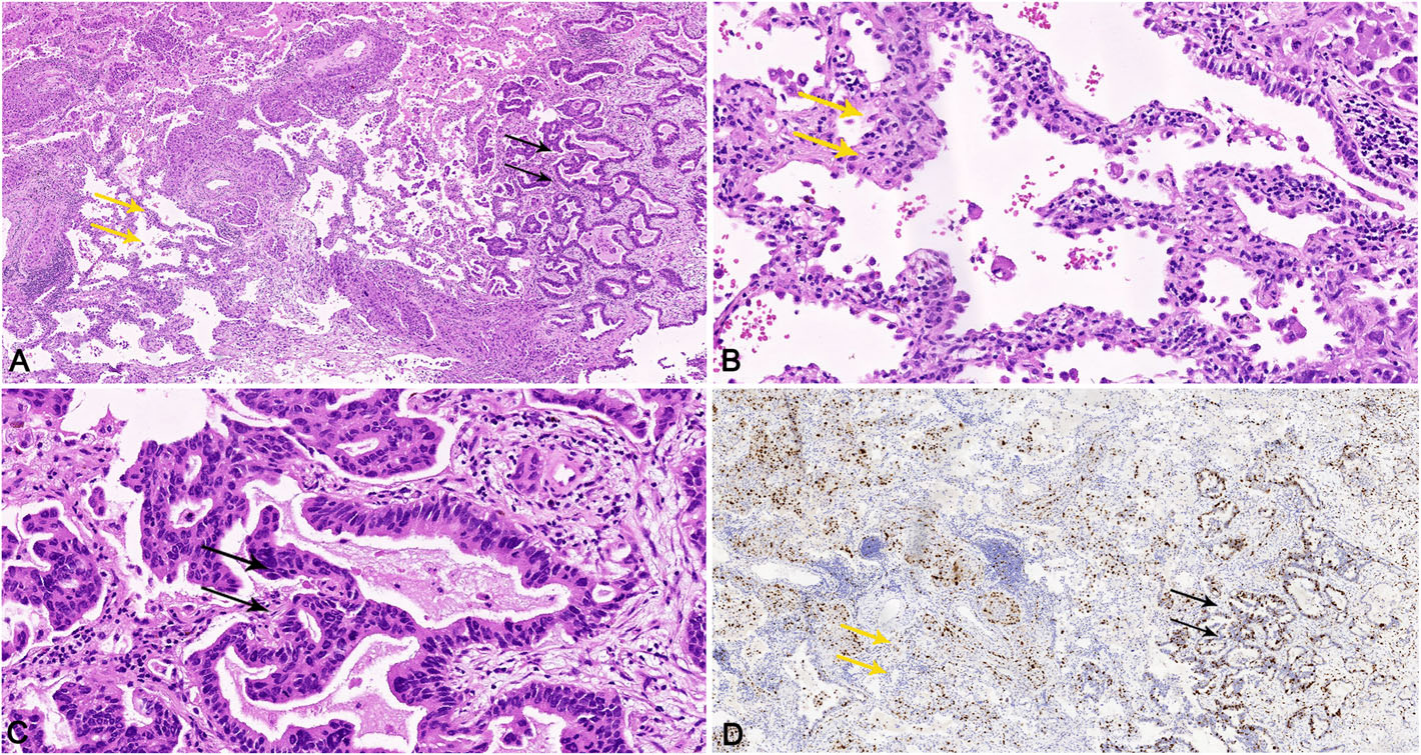
(**a**, **b**, **c**) Tumor nodule showed surface cells (yellow arrow) with atypical adenomatous hyperplasia transformed into adenocarcinoma (black arrow) (**h**&**e**). (**d**) Ki-67 proliferation index was significantly increased in malignant area (black area)

In the case reported, while most of the surface cells being similar to a typical PSP in some areas of the tumor, a few transformed to adenocarcinoma. The nuclei were columnar and containing hyperchromatic nuclear chromatin. In addition, the surface cells replaced the alveolar lining and invaded the fibrous stroma and vascular walls with TTF-1, EMA, Napsin A and CKpan all positive. The Ki-67 proliferation index was 70% (Fig. 3). We also noticed atypical adenomatous hyperplasia (AAH) of cuboidal cells in the transition area (Fig. 3). Cuboidal surface cells proliferated along preexisting alveolar walls with mild to moderate cellular atypia. A typical hobnail appearance was also seen in the atypical cuboidal surface cells. Substantial gaps along the surface of basement membrane in the transition area were also evident of AAH.

**Fig. 4:**
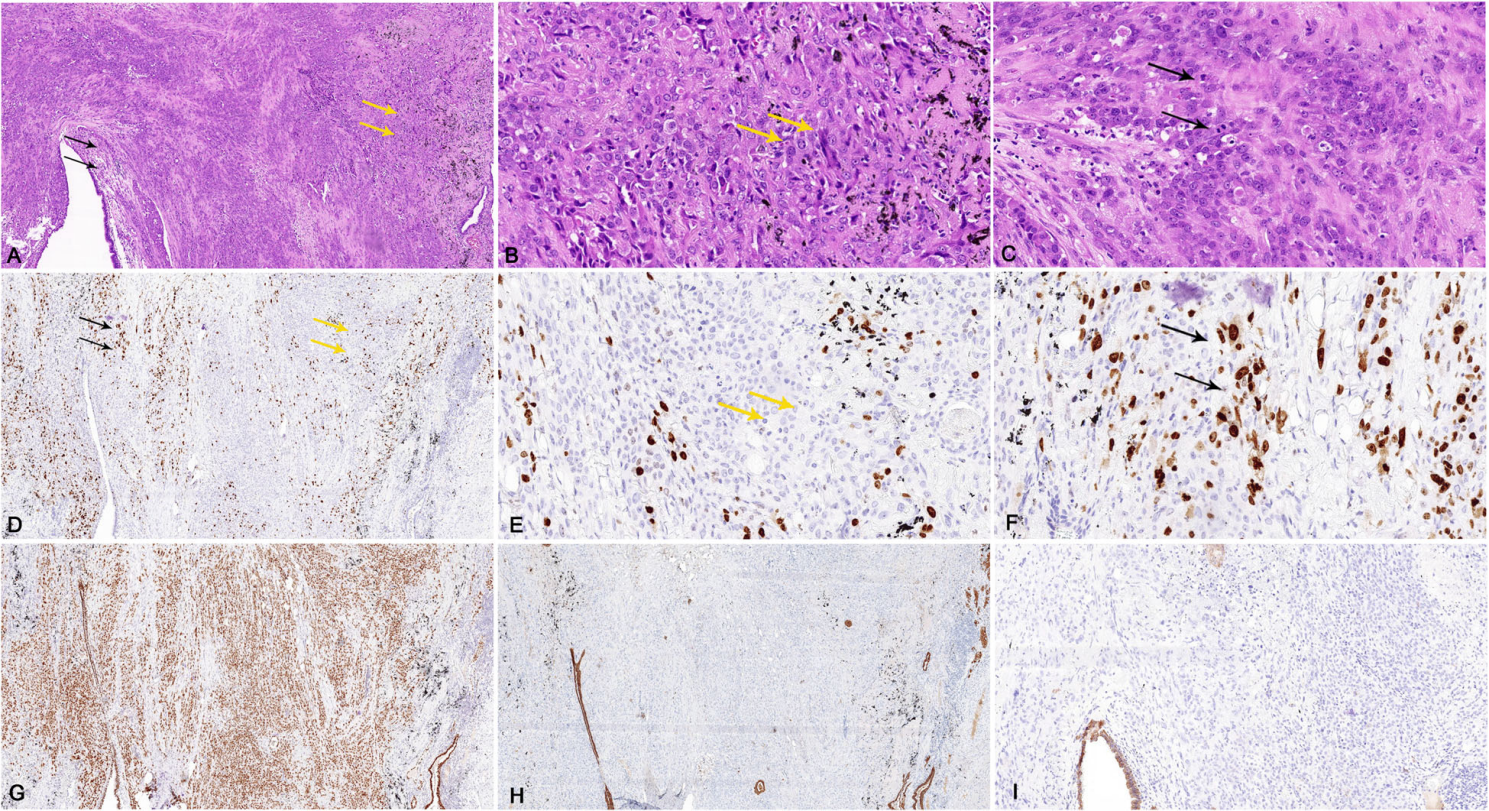
(**a**,**b**,**c**) Low power view and high power view demonstrated that stromal round cells in the left side transformed to the malignant tumor (black arrow), round cells in the transition area (yellow arrow) had dysplasia (**h**&**e**). (**d**, **b**, **f**) Low power view and high power view demonstrated that Ki-67 proliferation index was significantly increased in the malignant area (black arrow). (**g**) Thyroid transcription factor-1 (TTF-1) was positive in stromal round cells. (**h**) Pancytokeratin was negative in stromal round cells. (**i**) E-cadherin was negative in stromal round cells

A few stromal round cells had small, well-defined borders and central bland nuclei without nucleoli similar to that in a typical PSP. However, mild to moderate atypical stromal round cells proliferation was seen in the transition region (Fig. 4). Binuclearization and intranuclear eosinophilic inclusions were common in the transition area in our case. Furthermore, abundant cytoplasm, nuclear polymorphism, prominent nucleoli and irregular mitosis were observed in malignant stromal round cells, adjoining the transition areas (Fig. 4). Vascular invasion and pulmonary parenchyma involvements were also found in malignant lamellarlike stromal round cells. TTF-1, P63 and EMA were all positive. Only a small amount stromal round cells were positive for CKpan. However, stromal round cells were negative for beta-catenin and E-cadherin. The Ki-67 proliferation index in these areas was 55%, which was significantly increased compared to typical PSP areas (Fig. 4). Both stromal round cells and surface cells were negative for Progesterone receptor, CD20, CD3, S^− 100^, Melana, HMB45, Myogenin, MyoD1, CgA and Syn. Further molecular investigation using a polymerase chain reaction panel showed that no EGFR, ALK or ROS1 mutation was detected.

**Fig. 5:**
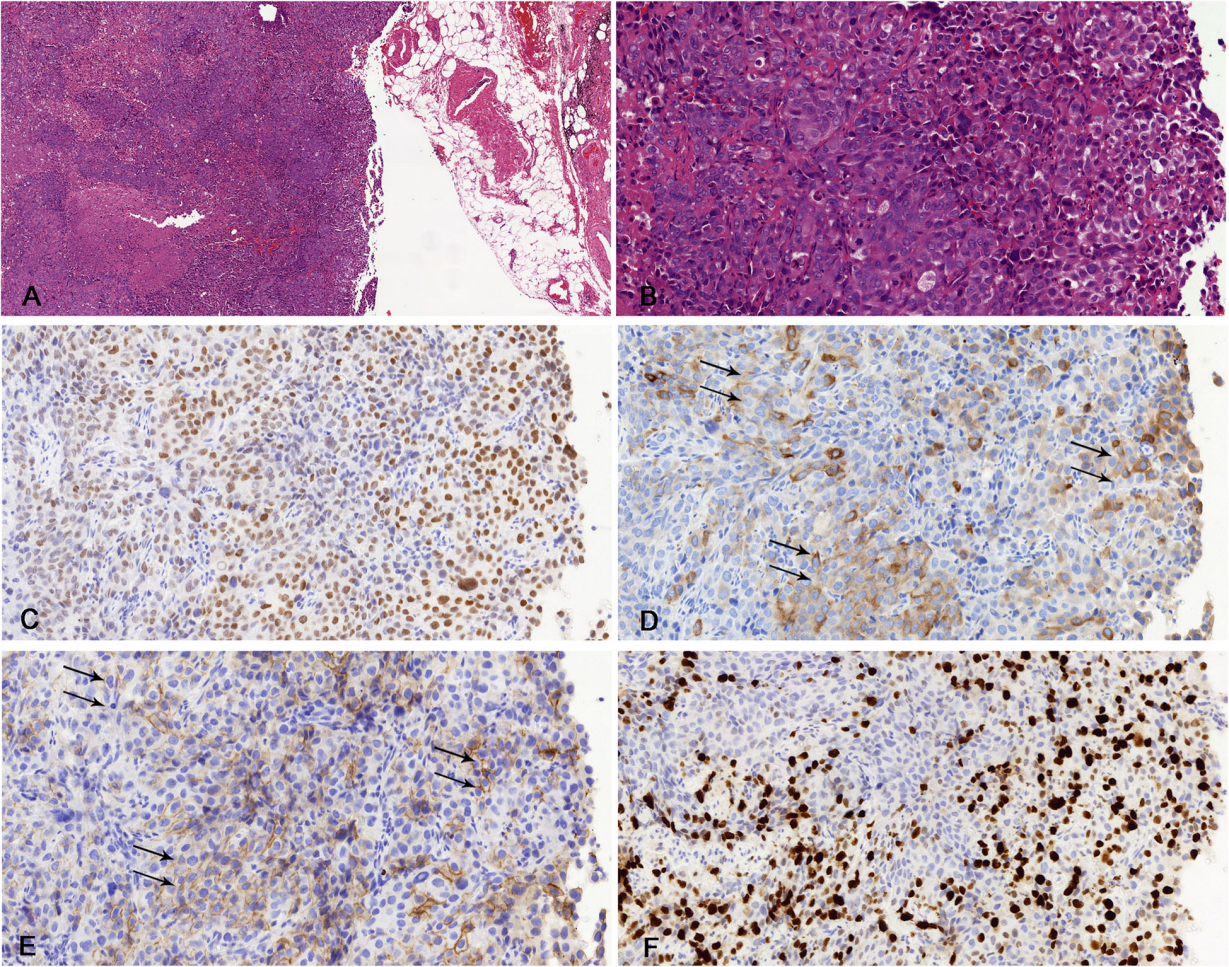
(**a**, **b**) Mediastinal lymph node metastasis of pulmonary sclerosing pneumocytoma (**h**&**e**). (**c**) Round cells were positive for thyroid transcription factor-1. (**d**) pancytokeratin (CKpan) were partial positive in round cells. (**e**) E-cadherin was partial positive in the metastatic lymph node. (**f**) Ki-67 proliferation index was significantly increased in the metastatic lymph node

In this case, we also found mediastinal lymph nodes involvement. The architecture of lymph nodes was replaced by abnormal proliferated stromal round cells with either vacuolated or eosinophilic cytoplasm (Fig. 5). IHC showed that these cells were positive for TTF-1, partial positive for CKpan and E-cadherin, but negative for beta-catenin. However, the E-cadherin was negative in malignant stromal round cells in the primary tumor (Fig. 4).

## Discussion and conclusion

PSP is considered as a rare benign tumor [1]. In searches of PubMed and Embase database, there are 24 cases of PSP with lymph node metastasis and recurrence (Table 1) [2, 6–25]. Five cases have mediastinal lymph node metastasis, four have distant metastasis, and only one have recurrence of PSP. However, no case about malignant transformation of PSP has been reported. One case reports overgrown stromal round cells and bone metastasis, accompanied by increased cellularity and necrotic areas, but a Ki-67 index of less than 5% [22]. Another case reports PSP with metastatic spread to stomach with Ki-67 indeice in primary tumor and metastatic gastric lesion of 17.6 and 19.4%, respectively [19]. The. However, no malignant pathomorphological change has been reported. In Iyoda’s research, cases with recurrence has a Ki-67 index of 0.4% [26]. These results show no significantly increased proliferation of cells even in patients with recurrence or metastasis. In our case, the Ki-67 proliferation index of the malignant cuboidal surface cells and the stromal round cells are 70 and 55%, respectively. The high proliferative activity and pathomorphological change in both cuboidal surface cells and stromal round cells suggest that PSP transformed to a malignant tumor. Liu reports a case of coexistence of PSP and primary adenocarcinoma in the same tumor [27], which is different from our case, where the AAH of cuboidal surface cells indicated malignant transformation from cuboidal surface cells to adenocarcinoma. Similarly, the malignant transformation of stromal round cells is also confirmed by the transition region.

**Table 1:** Studies of PSP with metastasis or recurrence

Author (Year)	Age	Gender	Primary location	Tumor size (mm)	Recurrence/ Metastatic site
Tanaka l (1986) [8]	22	Male	Right lower lobe	50	Hilar lymph node
Chan AC (2000) [9]	48	Male	Right lower lobe	80	Hilar lymph node
Devouassoux-Shisheboran M (2000) [2]	18	Female	Left lower lobe	35	Hilar lymph node
Yano M (2002) [10]	67	Female	Right lower lobe	90	Hilar lymph node
Kim KH (2003) [11]	19	Female	Left lower lobe	100	Hilum and intralobular lymph node
Kim GY (2003) [12]	37	Female	Left lower lobe	20	Supraclavicular lymph node
Miyagawa-Hayashino A (2003) [13]	10	Female	Right middle lobe	47	Regional lymph node
	45	Female	Right upper lobe	25	Hilar lymph node
	45	Male	Left lower lobe	37	Mediastinal lymph node
	50	Female	Left lower lobe	15	Intralobular lymph node
Chan NG (2003) [14]	19	Male	Left upper lobe	30	Intralobular lymph node
Katakura H (2005) [15]	35	Male	Left lower lobe	–	Mediastinal lymph node
Jiang ZN (2007) [16]	59	Female	Right lower lobe	65	Hilar lymph node
Wei S (2008) [9]	57	Female	Left lower lobe	25	Recurrence
Vaideeswar P (2009) [17]	23	Male	Right upper lobe	90	Hilar lymph node
Suzuki H (2011) [18]	57	Female	Right lower lobe	25	Pleural dissemination
Bae YS (2012) [19]	72	Female	Left lobe	32	Stomach
Kita H (2013) [20]	38	Female	Left lower lobe	39	Interlober lymph node
Adachi Y (2014) [6]	40	Female	Left lower lobe	10	Mediastinal lymph node
Xu HM (2015) [21]	26	Female	Right upper lobe	97	Hilar lymph node
Kim MK (2015) [22]	73	Female	Right lower lobe	35	Bone
Pokharel S (2016) [7]	33	Female	Left lower lobe	18	Mediastinal lymph node
Soo IX (2017) [23]	40	Female	Right lower lobe	25	Mediastinal lymph node
Wang X (2018) [24]	26	Female	Left lower lobe	40	Mediastinal and regional lymph nodes

In our case, the two well-established epithelial markers, E-cadherin and CKpan [28], are both positive in the metastatic lymph nodes with similar levels (black arrows in Fig. 5), although they should be negative in metastatic lymph nodes (composed of stromal round cells). The epithelial marker expression in metastatic lymph nodes suggests the mesenchymal-epithelial transition (MET) during lymph node metastasis. Previous studies show that MET process is able to promote distal metastasis in breast cancer [29], especially for establishing macrometastasis [30–32], which, combined with our results, suggests that the the MET process may play an important role in lymph node metastasis of PSP. Although based on previous studies, lymph node involvement doesn’t affect long-term survival rate [6, 23], patients with malignant PSP may still need close follow-up.

In summary, we report the first case of malignant transformation in both cuboidal surface cells and stromal round cells, which suggests the malignant potential of PSP. The fact that E-cadherin is negative in primary tumor but positive in metastatic lymph nodes suggests that the process of MET plays an important role in lymph node metastasis of PSP.

## Data Availability

The datasets used in this study are available from the corresponding author on reasonable requests.

## Abbreviations

AAH: Typical adenomatous hyperplasia
CKpan: Pancytokeratin
CT: Computed tomography
EMA: Epithelial membrane antigen
IHC: Immunohistochemistry
MET: Mesenchymal-epithelial transition
PSP: Pulmonary sclerosing pneumocytoma
TTF-1: Thyroid transcription factor-1
